# Effect of transvaginal *Lactobacillus* supplementation on reversing lower genital tract dysbiosis and improving perinatal outcomes in PCOS patients after IVF-FET: a study protocol for a multicenter randomized controlled trial

**DOI:** 10.1186/s13063-023-07825-9

**Published:** 2023-12-21

**Authors:** Huixi Chen, Yaoyao Tu, Chen Zhang, Jie Li, Ting Wu, Suying Liu, Liying He, Aijun Zhang, Yan Li, Lu Li, Yilun Sui, Li Wang, Xiaojun Chen, Ji Xi, Yanting Wu, Li Jin, He-Feng Huang

**Affiliations:** 1 Obstetrics and Gynecology Hospital, Institute of Reproduction and Development, Fudan University, Shanghai, China; 2 The International Peace Maternal and Child Health Hospital, School of Medicine, Shanghai Jiao Tong University, Shanghai, China; 3 Research Units of Embryo Original Diseases, Chinese Academy of Medical Sciences (No. 2019RU056), Shanghai, China; 4 Reproductive Center, Zhong Shan Hospital, Shanghai, China; 5 Reproductive Medical Center, Department of Obstetrics and Gynecology, Ruijin Hospital, Shanghai Jiao Tong University School of Medicine, Shanghai, China; 6 ShangHai JIAI Genetics and IVF Institute, Shanghai, China; 7 Key Laboratory of Reproductive Genetics (Ministry of Education), Department of Reproductive Endocrinology, Women’s Hospital, Zhejiang University School of Medicine, Hangzhou, China

**Keywords:** Study protocol, Polycystic ovary syndrome, Lower genital tract microbiome, Randomized controlled trial, Live *Lactobacillus* capsule for vaginal use, In vitro fertilization–frozen embryo transfer, Clinical pregnancy, Live birth

## Abstract

**Background:**

Significant lower genital tract (LGT) dysbiosis and an associated lower rate of clinical pregnancy after in vitro fertilization–frozen embryo transfer (IVF-FET) among polycystic ovary syndrome (PCOS) patients have been previously reported by our group. We aimed to assess whether transvaginal *Lactobacillus* supplementation can reverse LGT dysbiosis and further improve perinatal outcomes in PCOS patients after IVF-FET.

**Methods/design:**

This is a protocol for a multicenter, open-label, randomized controlled trial in China. Women diagnosed with PCOS who are undergoing IVF-FET treatment will be recruited. Allocation to the intervention/control arms at a ratio of 1:1 will be executed by an electronic randomization system. Participants in the intervention arm will receive the live *Lactobacillus* capsule vaginally for 10 consecutive days before embryo transfer, while those in the control arm will receive standard individualized care. The primary outcomes will be the clinical pregnancy rate, implantation rate, and live birth rate. 16S rRNA sequencing and liquid chromatography–mass spectrometry will be conducted to evaluate the LGT microbiome and systemic metabonomics before and after the intervention. A sample of 260 participants will provide 95% power to detect a 20% increase in the rate of clinical pregnancy (*α* = 0.025, one-tailed test, 15% dropout rate). A total of 300 participants will be recruited.

**Discussion:**

This is the first large and multicenter randomized controlled trial aimed at assessing the efficacy of transvaginal *Lactobacillus* supplementation on restoring the LGT microbiome and improving perinatal outcomes in PCOS patients after IVF-FET. This pragmatic trial is promising for increasing the rates of clinical pregnancy and live birth in PCOS patients after IVF-FET.

**Ethics and dissemination:**

Ethical review approval was obtained from the Medical Research Ethics Committees of the International Peace Maternity and Child Health Hospital of Shanghai Jiao Tong University (15 October 2020, GKLW 2020-29). To maximize dissemination, these findings will be reported in open access publications in journals with high impact, and oral and poster conference presentations will be performed.

**Trial registration:**

ChiCTR ChiCTR2000036460. Registered on 13 September 2020, https://www.chictr.org.cn/showproj.html?proj=59549.

**Supplementary Information:**

The online version contains supplementary material available at 10.1186/s13063-023-07825-9.

## Background

Polycystic ovarian syndrome (PCOS) is one of the most common endocrinopathies among women of reproductive age, affecting approximately 10–15% of women worldwide, and is one of the most common causes of female infertility [1, 2]. According to the Rotterdam criteria, two of the following three criteria must be fulfilled for a PCOS diagnosis: hyperandrogenism, oligo-ovulation or anovulation, and polycystic ovarian morphology defined by the presence of ≥ 12 follicles with a maximum diameter of 2–9 mm in either ovary [3, 4]. Women with PCOS are at a high risk of infertility due to anovulation. In vitro fertilization–frozen embryo transfer (IVF-FET) provides a valuable choice for PCOS patients who desire to become pregnant. However, once pregnant, women with PCOS are more susceptible to recurrent miscarriage, gestational diabetes mellitus (GDM), hypertension disorders in pregnancy, etc. [5, 6].

Our previous study showed that the bacterial genus diversity was higher in the lower genital tract (LGT), including the vagina and cervical canal, in PCOS patients. Additionally, the abundance of *Lactobacillus* spp. was significantly reduced in the LGT of PCOS patients. Pathway enrichment analysis showed that the top pathways were beneficial to the growth of pathogenic species, including *Gardnerella* [7]. Importantly, we found that in PCOS patients undergoing IVF-FET, those with *Lactobacillus* deficiency in the LGT had significantly lower rates of embryo implantation and clinical pregnancy (unpublished data), suggesting a potential role of reversing LGT dysbiosis in improving perinatal outcomes in PCOS patients after IVF-FET. However, large-scale and multicenter randomized clinical trials (RCTs) examining the effect of a vaginal *Lactobacillus* intervention on improving pregnancy outcomes in PCOS patients with LGT flora dysbiosis are unavailable.

The use of probiotics mainly containing *Lactobacillus* spp. was previously reported to have benefits in the treatment of vaginal diseases. A recent meta-analysis (*n* = 2321) showed that probiotic therapy resulted in a beneficial outcome in the treatment of bacterial vaginosis [8]. Compared with antibiotic therapy for bacterial vaginosis, which has high failure and recurrence rates, probiotics are recognized as valuable alternative strategies for their significant role in reestablishing the physiological vaginal environment and improving the local immune response with fewer adverse effects, further relieving clinical symptoms [9]. Moreover, a meta-analysis systematically reviewed the beneficial outcomes of probiotic treatment for women with GDM, including maternal and infant health and well-being [10].

PCOS patients were reported to have metabolic disorders, mainly hyperandrogenism, insulin resistance, dyslipidemia, and derangements in carbohydrate and hormonal metabolism [4, 11–15]. Moreover, studies reported that metabolic profiling and the vaginal microbiome were associated with adverse perinatal outcomes such as preterm birth [16]. However, the changes in metabonomics in the LGT and their interactions with systemic metabolic status in PCOS patients remain unknown, as do their effects on IVF–FET and maternal and infant health outcomes. Given the adverse perinatal effects of LGT dysbacteriosis on PCOS patients and the promising benefits of reversing LGT dysbacteriosis on improving the perinatal outcomes of PCOS patients, we designed this multicenter, open-label RCT to provide an accessible and effective therapy to better improve the perinatal outcomes and health of PCOS patients with LGT dysbiosis who are undergoing IVF–FET.

## Methods/design

### Objectives

We plan to conduct the present trial to evaluate the effects of supplementation with live *Lactobacillus* capsules for vaginal use on reversing LGT dysbiosis and improving perinatal outcomes in PCOS patients undergoing IVF-FET.

### Study design and setting

**Fig. 1:**
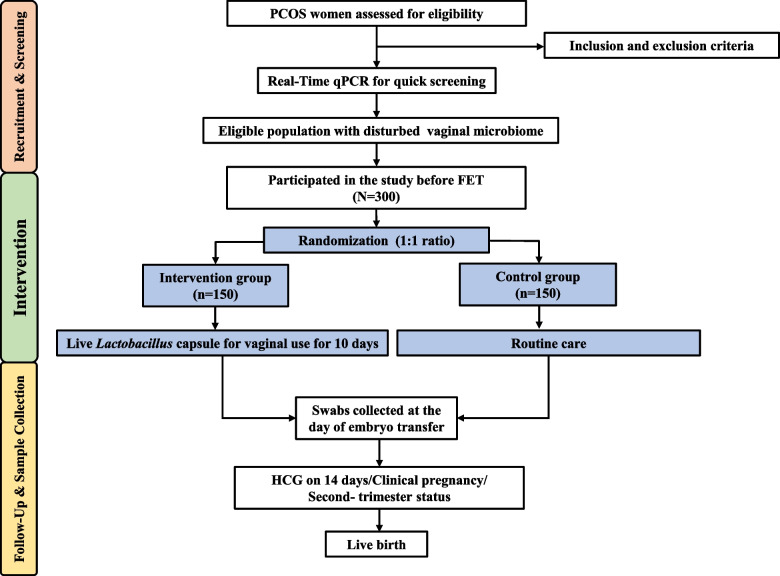
Flowchart for recruitment, screening, allocation, intervention, and follow-up of study participants. PCOS, polycystic ovary syndrome; qPCR, quantitative polymerase chain reaction; FET, frozen embryo transfer; HCG, human chorionic gonadotropin

This proposed study is a prospective, multicenter, open-label RCT. A total of 300 eligible participants will be recruited from 4 different tertiary hospitals, including ± 100 participants from the International Peace Maternity and Child Health Hospital, ± 50 participants from Ruijin Hospital, ± 50 participants from Zhongshan Hospital, and ± 100 participants from ShangHai JIAI Genetics and IVF Institute. A flowchart of participant enrollment and screening, randomization, and follow-up and sample collection is shown in Fig. 1.

### Participants

#### Inclusion criteria

A total of 20- to 35-year-old women who have been diagnosed with PCOS according to the Rotterdam Criteria and plan to undergo IVF–FET will be recruited. Quick screening of the abundance of *Lactobacillus* spp. will be performed by real-time quantitative polymerase chain reaction (RT–qPCR), which will be specified later. Women with PCOS with *Lactobacillus* spp. accounting for less than 80% of the vaginal microbiome who volunteer to participate will be eligible for this study.

#### Exclusion criteria

We will exclude individuals if they have been diagnosed with the following disorders: Cushing’s syndrome, congenital adrenal hyperplasia, thyroid disorder, hyperprolactinemia, androgen-secreting tumors, congenital malformations of the reproductive tract, systemic lupus erythematosus or any other autoimmune disease, HIV, endometriosis, uterine fibroids or colpitis. The subjects should not receive hormones, antibiotics, or vaginal medication within 7 days or cervical treatment or flushing within 5 days of the start of the study. Moreover, sexual activities should not be carried out within 48 hours. Women who undergo preimplantation genetic testing will also be excluded.

### Withdrawal of participants

Participants will be clearly informed by investigators at enrollment that they may withdraw from this RCT at any period for any reason without penalty. A withdrawal statement will be provided and signed. Written consent forms will be obtained if the individual agrees to receive continued follow-up for associated clinical outcomes after withdrawal.

### Procedure

The study procedure is described by following the Standard Protocol Items: Recommendations for Interventional Trials (SPIRIT) checklist (Additional file 1).

### Recruitment and randomization

The clinical research coordinators (CRCs) will verbally inform PCOS patients that the hospital is currently conducting a multicenter clinical trial to better improve the outcomes of IVF–FET in PCOS patients with vaginal microbiota dysbiosis. If they fully understand and are willing to take part in the study, they will meet a CRC in person at the hospital, sign an informed consent form, and officially join the study on the first day of the current IVF–FET cycle. The CRCs will later inform patients of whether they have vaginal microbiota dysbiosis. Basic information, including sociodemographic characteristics and clinical history, will be collected by the CRCs using standardized questionnaires and recorded into an online and password-protected electronic data collection system (Clinical Research Integrated Platform [CRIP], https://crip-ec.shdc.org.cn, supported by Shanghai Hospital Development Center, Shanghai, China), which will automatically generate a sequential code and randomly allocate participants to the intervention group or the control group at a ratio of 1:1 based on the whole study population. The research doctors will manage the study group allocation. Given the nature of vaginal use of live *Lactobacillus* capsules before FET, the investigators and participants will be informed of the assignment. To ensure the reality of this study, independent statisticians will be recruited, and they will be blinded to the group allocation.

### Sample collection

Two vaginal swabs, 2 cervical canal swabs, and 3 ml of peripheral blood will be collected on the day of the first clinic visit and the day of embryo transfer (ET). Vaginal swabs will be collected directly, while cervical canal swabs will be collected carefully through a vaginal dilator device to avoid contamination from contact with the vagina. Vaginal and cervical canal swabs will be immediately collected in a clean 2-ml DNA LoBind tube and immediately placed on ice and transferred to a − 80 °C freezer within 2 h, followed by DNA extraction. Peripheral venous blood will be collected in coagulation-enhancing disposable vacuum blood collection tubes. Blood will be centrifuged at 2000 rpm for 20 min at 4 °C (Eppendorf, Germany). Serum will be isolated and divided into 2 tubes and immediately frozen at − 80 °C until use.

### DNA extraction and quick screening by quantitative PCR

DNA extraction from a vaginal swab will be performed using the DNeasy Power Soil kit (12888, QIAGEN) according to the manufacturer’s instructions. The quantity and quality of DNA will be assessed by a NanoDrop 2000 (Thermo Fisher Scientific, USA). Quick screening for the relative abundance of *Lactobacillus* spp. in the vaginal microbiome will be performed by qPCR. The relative abundance of *Gardnerella vaginalis* and subtypes of *Lactobacillus* spp., including *Lactobacillus jensenii*, *Lactobacillus crispatus*, and *Lactobacillus ineris,* will also be evaluated. The sequences of primers used in qPCR are presented in Table 1. DNA will be amplified with TB Green® Premix Ex Taq™ (Tli RNaseH Plus) (RR420, Takara) using an ABI QuantStudio™ 7 Flex Real-Time PCR System. The abundance of *Lactobacillus* spp. will be calculated relative to that of total bacteria.

**Table 1:** List of primers used for qPCR

Target	Forward	Reverse
Total bacteria	5′-AGAGTTTGATCCTGGCTCAG-3′	5′-GCTGCCTCCCGTAGGAGT-3′
*Lactobacillus* spp.	5′-AGAGTTTGAT/YM/TGGCTCAG-3′	5′-CACCGCTACACATGGAG-3′
*Lactobacillus jensenii*	5′-AAGTCGAGCGAGCTTGCCTATAGA-3′	5′-CTTCTTTCATGCGAAAGTAGC-3′
*Lactobacillus crispatus*	5′-AGCGAGCGGAACTAACAGATTTAC-3′	5′-AGCTGATCATGCGATCTGCTT-3′
*Lactobacillus ineris*	5′-CTCTGCCTTGAAGATCGGAGTGC-3′	5′-ACAGTTGATAGGCATCATCTG-3′
*Gardnerella vaginalis*	5′-TTACTGGTGTATCACTGTAAGG-3′	5′-CCGTCACAGGCTGAACAGT-3′

### Intervention

For the intervention group, a live *Lactobacillus* capsule (> 1 × 10^6^ CFU/g, National Medicine Approval Number: S20030005, Inner Mongolia Shuangqi Pharmaceutical Co., Ltd., PRC) will be administered vaginally.

The live *Lactobacillus* capsule has been reported to be effective for bacterial vaginosis treatment with fewer adverse effects than antibiotics, and probiotic treatment for women with GDM has been reported to improve maternal and infant health outcomes [10, 17–19]. In the intervention group, 1 live *Lactobacillus* capsule per day will be applied transvaginally for 10 consecutive days starting on the initiation day of the FET cycle. Medical gloves will be included in the pill box, and the pills will be stored at 2–8 °C until use. The usage will be explained to the participants by the CRCs. A well-designed medication diary will be prepared to help remind patients and assist them in recording the process of medication administration (Table 2). Regular follow-up will be performed on day 5 and day 10 in both groups by phone call. Any adverse response will be recorded and reported at any time.

**Table 2:** Medication diary

Medication diary			
Name:			
Date	Day Month Year	Day Month Year	Day Month Year
Live *Lactobacillus* capsule for vaginal use	□ Morning □ Evening ______pill	□ Morning □ Evening ______pill	□ Morning □ Evening ______pill
	Day Month Year	Day Month Year	Day Month Year
	□ Morning □ Evening ______pill	□ Morning □ Evening ______pill	□ Morning □ Evening ______pill
	Day Month Year	Day Month Year	Day Month Year
	□ Morning □ Evening ______pill	□ Morning □ Evening ______pill	□ Morning □ Evening ______pill
	Day Month Year		
	□ Morning □ Evening ______pill		

Please fill out the date and time after the daily use of 1 capsule

Please contact us if any symptoms of discomfort occur during medication use

### Standard care

All participants in both groups will receive optimally personalized endometrial preparation regimens. The medication regimens will be recorded in detail, including the dose, frequency, and duration of use of the medication.

### Participant timeline

The time schedule of enrollment, intervention, follow-up and outcome assessments is shown in Table 3. After undergoing ovulation, egg retrieval, IVF, and advanced embryo freezing, PCOS patients will move on to the next step of endometrial preparation for FET. The duration of endometrial preparation will continue for approximately 14 days. At the 1st clinical visit on the first day of the current FET cycle, written informed consent, baseline information, 2 vaginal swabs, 2 cervical canal swabs, and 3 ml peripheral blood will be obtained at enrollment. After quick screening for the relative abundance of *Lactobacillus* spp. in vaginal flora, the participants will be informed of whether they have LGT dysbiosis and the results of randomization. At the 2nd visit after enrollment, in the control group, participants with LGT dysbiosis will confirm the next contact, while the intervention group will administer live *Lactobacillus* capsules vaginally and will be provided a medication diary. All participants will be followed up 5 times in the clinic or by phone call. The 3rd visit after 5 days of drug administration will mainly be about whether any adverse response occurs. At the 4th visit after 10 days of drug administration, the medication diary will be checked and reviewed, and any adverse response will be recorded and reported. At the 5th visit on the day of ET, 2 vaginal swabs, 2 cervical canal swabs, and 3 ml peripheral blood will be obtained from participants in both groups. At the 1st follow-up after 14 days of ET, human chorionic gonadotropin (HCG) and progesterone (P) levels will be determined as standard clinical procedures. If the HCG test is positive, the 2nd follow-up will occur approximately 5 weeks after ET, and clinical pregnancy will be confirmed by ultrasound and recorded in the CRIP system. At the 3rd follow-up, the fetal developmental situation and maternal obstetric complications at approximately 24-28 gestational weeks, if any, will be carefully recorded. The endpoint of follow-up is live birth, and maternal and neonatal information will be obtained.

**Table 3:** Schedule

	1st visit on FET initial day	2nd visit after enrollment	3rd visit after 5 days	4th visit after 10 days	5th visit on ET	1st follow-up at 14 days after ET	2nd follow-up at 5 weeks after ET	3rd follow-up at mid-pregnancy	End of live birth
**Enrollment**									
Eligibility screen	**X**								
Written informed consent	**X**								
Allocation		**X**							
**Interventions**									
Optimal endometrial preparation	**X**	**X**	**X**	**X**	**X**				
*Lactobacillus* capsule		**X**	**X**	**X**					
**Assessments**									
Baseline questionnaire	**X**								
Vaginal and cervical canal swabs	**X**				**X**				
Peripheral blood samples	**X**				**X**				
Medication diary (intervention group)		**X**	**X**	**X**					
HCG and P						**X**	**X**		
Clinical pregnancy							**X**		
Maternal and Fetal information							**X**	**X**	**X**
Safety assessment		**X**	**X**	**X**	**X**	**X**	**X**	**X**	**X**

*HCG*, human chorionic gonadotropin; *P*, progesterone

### 16S rRNA amplicon sequencing

For the analysis of bacterial diversity, V3-V4 variable regions of 16S rRNA genes will be amplified with universal primer pairs 343F (5′-TACGGRAGGCAGCAG-3′) and 798R (5′-AGGGTATCTAATCCT-3′) [20]. 16S rRNA sequencing will be performed on an Illumina NovaSeq 6000 with 250 bp paired-end reads (Illumina Inc., San Diego, CA; OE Biotech Company; Shanghai, China).

### Liquid chromatography–mass spectrometry (LC–MS) for metabonomic analysis

An ACQUITY UPLC I-Class system (Waters Corporation, Milford, USA) coupled with a VION IMS QTOF mass spectrometer (Waters Corporation, Milford, USA) will be used to analyze the metabolic profile in both ESI positive and ESI negative ion modes.

### Proposed outcome measurement

#### Primary outcome

The clinical pregnancy rate will be the primary outcome. The clinical pregnancy rate is defined as the ratio of the clinical pregnancy (one or more observed gestational sacs with a fetal heartbeat in the uterus under ultrasonography 28 days after embryo transfer) to the number of transplant cycles.

#### Secondary outcomes

##### Maternal outcomes

The implantation rate is defined as the number of gestational sacs in the uterus divided by the number of embryos transferred. The live birth rate is defined as the delivery of any viable infant at 25 weeks of gestation or more after initial embryo transfer to the number of transplant cycles. The biochemical pregnancy rate is defined as the ratio of biochemical pregnancies (serum hCG level >5 mIU/L 14 days after embryo transfer) to the number of transplant cycles. The spontaneous abortion rate is defined as the ratio of spontaneous abortions (spontaneous pregnancy loss before 20 weeks of gestation) to the number of transplant cycles. The ongoing pregnancy rate is defined as the ratio of ongoing pregnancies (intrauterine live fetus under ultrasonography after 20 weeks of gestation) to the number of transplant cycles. The preterm birth rate is defined as the ratio of preterm births (delivery at ≥ 28 and < 37 weeks of gestation) to the number of transplant cycles. Pregnancy complications, including GDM, hypertensive disorders of pregnancy, including gestational hypertension and preeclampsia, placental abruption, preterm/prelabor/rupture of membranes, birth mode (vaginal delivery/operational vaginal delivery/selective or emergency cesarean section), blood loss during birth, and postpartum hemorrhage, adherence to the intervention and maternal mortality, will be followed up and assessed. A GDM diagnosis will be made according to the diagnostic criteria of the American Diabetes Association based on the results of the 75-g oral glucose tolerance test (OGTT) at 24–28 gestational weeks (fasting venous glucose level ≥ 5.1 mmol/L, 1-h value ≥ 10.0 mmol/L, 2-h value ≥ 8.5 mmol/L) [21]. Gestational hypertension is defined as de novo hypertension (≥ 140/90 mmHg) after 20 gestational weeks without proteinuria. Preeclampsia will be diagnosed when gestational hypertension is comorbid with any of the following manifestations: proteinuria (≥ 0.3 g/24 h), renal insufficiency, thrombocytopenia (platelets ≤ 100,000 × 10^9^/L), impaired liver function or pulmonary edema [22].

##### Neonatal outcomes

The birth defect rate is defined as the ratio of birth defects (fetal body structure, functional or metabolic congenital abnormalities) to the number of transplant cycles*.* Birth weight, infant sex, gestational age at birth, Apgar score, congenital anomalies, shoulder dystocia, bone fracture, neonatal hypoglycemia, bronchial plexus injury, neonatal respiratory distress, neonatal pathological jaundice, neonatal intensive care unit admission, stillbirth (≥ 20 weeks), and neonatal death (within the first 28 days after delivery) will be followed-up and assessed.

### Microbiome and metabolism

Alpha and beta diversity analysis will be conducted by QIIME2 software [23]. The taxonomy abundance spectrum will be described and compared between groups using the linear discriminant analysis effect size (LEfSe) method. Metabolic profiles will be analyzed using the Progenesis QI v3.0 software.

### Data collection

At the time of enrollment, information about the history of PCOS diagnosis, menstrual cycle demographic characteristics, socioeconomic status, alcohol consumption, smoking habit, self-reported morbidity, obstetric history, first-degree family history of hypertension, diabetes, stroke, hyperlipidemia, and obesity will be obtained using a baseline questionnaire. Clinical information on the maternal and neonatal outcomes mentioned above will be collected from outpatient and inpatient electronic records in four centers and will be confirmed by the investigators.

### Statistical methods

#### Sample size calculation

A recent meta-analysis reported an estimated clinical pregnancy rate of 26% in PCOS patients undergoing IVF [24]. Our team has revealed that approximately 30% of PCOS patients have *Lactobacillus* deficiency (PCOS-LD), which presents as lower levels of *Lactobacillus* (< 50%) in the vagina [7]. Our unpublished data suggested a clinical pregnancy rate of 17.65% in patients with PCOS-LD, while a clinical pregnancy rate of 41.82% was reported in PCOS patients without *Lactobacillus* deficiency (PCOS-non-LD). Moreover, our team previously reported a clinical pregnancy rate of 49.69% in patients who underwent IVF due to tubal blockage [25]. To improve the clinical pregnancy rate among infertile PCOS-LD patients to the level of that among PCOS-non-LD patients, or even to the clinical pregnancy rate among patients with tubal blockage, we hypothesized a 20% increase in the clinical pregnancy rate in our RCT. Assuming a clinically important 20% increase in the rate of clinical pregnancy in the intervention arm and allowing for a 15% dropout rate, the calculated sample size of 260 subjects (130 per group) will provide 95% power with a one-tailed alpha error of 0.025. Thus, we aim to recruit 300 women in total (150 per group).

#### Statistical analysis

Categorical variables will be presented as counts with percentages, e.g., *n* (%). Normally distributed data will be shown as the means with standard deviations, e.g., means ± SDs, while nonnormally distributed data will be expressed as medians with interquartile ranges, e.g., medians (IQRs). Student’s *t* test for continuous variables with normal distributions and the nonparametric

Kruskal–Wallis test for nonnormally distributed data will be applied for comparisons of significant differences between the 2 groups. The Pearson chi-square test will be used for categorical data. For binary observational endpoints, risk ratios with 95% confidence intervals will be calculated by binomial regression with and without adjustment for potentially confounding baseline values. In this study, the main analyses will be conducted using an intention-to-treat (ITT) model, in which all participants will be included based on random assignment regardless of study completion. For outcomes collected at different time points, a repeated measurements design using a generalized estimating equation (GEE) model will be used to fit outcomes that are measured at each time point so that all the participants might be included in the analyses, even if there are missing data for some of the follow-up points. A multiple imputation model will be used to process the missing data. Additionally, statistical analyses of the baseline characteristics of participants who remain in the study and those who are lost to follow-up will also be performed to explore whether there is differential dropout. All statistical analyses will be conducted using the SPSS software (version 25.0) and R software (version 4.0.2). A two-sided *p* value < 0.05 will be considered significant.

### Data management and confidentiality

All paper materials will be stored and locked in a designated cabinet, and electronic data will be saved in the password-secured CRIP system. Data input will be executed by a full-time CRC and a supervisor. Incorrectly inputted data will be checked and corrected by the supervisor after confirmation with participants or by checking their clinical records. Any amendment of the original data will be tracked in detail. The personal information of the participants will remain anonymous and be stored securely throughout the study. Any unauthorized access to the CRIP system or disclosure of the database will not be allowed at any trial center. All staff members in this study are responsible for strict confidentiality of information at all times. There are no plans for additional studies using the data that are collected as part of this trial.

### Data monitoring and auditing

The trial steering committee is composed of LL, SYL, AJZ, HFH, and JL, who will be responsible for study supervision and overall conduct. A data monitoring committee (DMC) will be set up for on-site audits and quality control. The DMC will be independent from the sponsor with no conflicts of interest. Two audits will be executed: at the midpoint and at the end of the RCT. Emphasis will be placed on whether enrollment, sampling, data collection, intervention, and follow-ups are executed and recorded in a timely manner according to the research protocol. The accuracy of the original data will be verified by randomly selected participants. A formal report will be generated, which will provide valuable instructions for problem-solving in due time and for future statistical analyses. Regarding adverse events (AEs), vaginal use of live *Lactobacillus* capsules for the treatment of mycosis fungoides and bacterial vaginosis in pregnant women has been reported, and no adverse effects were observed in a small-scale clinical study in China [17–19]. However, for the safety of the treatment, during the whole treatment and follow-up procedure, any discomfort or worsening of existing discomfort in patients will be recorded, regardless of whether it is related to the study intervention. Symptoms such as abdominal pain, bloating, chest tightness, and bleeding, physical signs such as positive shifting dullness, adnexa mass, and infection and abnormal medical auxiliary examination results will be recorded. Any unexpected effects might be collected by regular follow-up, participant self-reports, systematic physical examinations performed by doctors, and laboratory results. Any harms will be classified using the Common Terminology Criteria for Adverse Events (CTCAE) Version 5, published on November 27, 2017, by the Department of Health and Human Services, National Institutes of Health, National Cancer Institute, USA. Any unintended appearances of AEs will be reported in a timely manner to the TSC and recorded in detail. All the related AEs will be recorded in case report form and reported in the future published manuscript.

### Ethics approval and consent to participate

Ethical review approvals were obtained from four centers: the International Peace Maternity and Child Health Hospital (15 October 2020, GKLW 2020-29), Ruijin Hospital, Shanghai Jiao Tong University, School of Medicine, Shanghai, China (3 December 2020), Zhongshan Hospital, Fudan University, Shanghai, China (20 October 2020, SK2020-169), and the ShangHai JIAI Genetics and IVF Institute (10 October 2020, JIAI E2020-017). All study designs will comply with the principles of the Declaration of Helsinki. All individuals will sign written informed consent forms before participating in the RCT.

### Dissemination

Regular meetings will be held for quality control and progress promotion. The completion of this trial and publication of the results will be attributed to all collaborators. The ultimate results of this trial will be reported back to each participant through open access publications. To maximize dissemination, these findings will be reported in open access publications in journals with high impact, and oral and poster presentations concerning the fertility needs of PCOS patients will be performed domestically and internationally.

## Discussion

PCOS is one of the most common causes of female infertility. Once pregnant, women with PCOS face an increased risk of adverse perinatal outcomes [5]. Moreover, we previously found significant LGT dysbiosis and an associated lower rate of clinical pregnancy among PCOS patients after IVF-FET (unpublished). Our trial aims to evaluate the effects of transvaginal *Lactobacillus* supplementation on the health of the LGT microbiome and perinatal outcomes in PCOS patients after IVF-FET, which will be the first large and cross-centered RCT in this territory. If successful, the findings of this study can be applied to clinical practice to better improve the health of PCOS patients and maternal and infant health after IVF-FET.

Nonetheless, several limitations of this trial should be recognized. First, investigators and participants will not be blinded to group allocation, which might introduce bias. Another limitation is that the control arm will still have access to the administration of *Lactobacillus*, although medical records will be obtained to confirm the group situation.

## Trial status

Recruitment began on January 12, 2021. Data collection aims to be completed in July 2024. Date and version identifier: 2020.08.15-version 1.0.

### Supplementary Information

**Additional file 1.** SPIRIT 2013 Checklist: Recommended items to address in a clinical trial protocol and related document.

## Data Availability

Not applicable.

## Abbreviations

PCOS: Polycystic ovary syndrome
LGT: Lower genital tract
IVF-FET: In vitro fertilization–frozen embryo transfer
GDM: Gestational diabetes mellitus
RCT: Randomized clinical trial
RT–qPCR: Real-time quantitative polymerase chain reaction
CRCs: Clinical research coordinators
CRIP: Clinical Research Integrated Platform
ET: Embryo transfer
HCG: Human chorionic gonadotropin
P: Progesterone
LC–MS: Liquid chromatography–mass spectrometry
LEfSe: Linear discriminant analysis effect size
SDs: Standard deviations
IQRs: Interquartile ranges
TSC: Trial steering committee
DMC: Data monitoring committee
AEs: Adverse events
